# A Cost Effective Scheme for the Highly Accurate Description of Intermolecular Binding in Large Complexes

**DOI:** 10.3390/ijms232415773

**Published:** 2022-12-12

**Authors:** Jiří Czernek, Jiří Brus, Vladimíra Czerneková

**Affiliations:** 1 Institute of Macromolecular Chemistry, Czech Academy of Sciences, Heyrovsky Square 2, 162 00 Prague, Czech Republic; 2 Institute of Physics, Czech Academy of Science, Na Slovance 2, 182 21 Prague, Czech Republic

**Keywords:** noncovalent interactions, intermolecular binding, CCSD(T), DLPNO, DFT-SAPT

## Abstract

There has been a growing interest in quantitative predictions of the intermolecular binding energy of large complexes. One of the most important quantum chemical techniques capable of such predictions is the domain-based local pair natural orbital (DLPNO) scheme for the coupled cluster theory with singles, doubles, and iterative triples [CCSD(T)], whose results are extrapolated to the complete basis set (CBS) limit. Here, the DLPNO-based focal-point method is devised with the aim of obtaining CBS-extrapolated values that are very close to their canonical CCSD(T)/CBS counterparts, and thus may serve for routinely checking a performance of less expensive computational methods, for example, those based on the density-functional theory (DFT). The efficacy of this method is demonstrated for several sets of noncovalent complexes with varying amounts of the electrostatics, induction, and dispersion contributions to binding (as revealed by accurate DFT-based symmetry-adapted perturbation theory (SAPT) calculations). It is shown that when applied to dimeric models of poly(3-hydroxybutyrate) chains in its two polymorphic forms, the DLPNO-CCSD(T) and DFT-SAPT computational schemes agree to within about 2 kJ/mol of an absolute value of the interaction energy. These computational schemes thus should be useful for a reliable description of factors leading to the enthalpic stabilization of extended systems.

## 1. Introduction

A quantitative description of intermolecular noncovalent interactions is the key factor in understanding properties of gaseous and condensed phases [1], molecular recognition [2], some chemical transformations [3], and supramolecular structures [4]. Hence, intermolecular interactions are intensely studied by both experimental and theoretical methods, as reviewed in reference [5]. From among of these theoretical methods, of particular importance is the symmetry adapted perturbation theory (SAPT) of intermolecular interactions [6], since it can be combined with the density-functional theory (DFT) treatment of monomers [7] to accurately characterize the nature of noncovalent bonding even for very large (containing over 100 atoms) complexes [8,9,10]; this technique is denoted here as DFT-SAPT. Another particularly important group of theoretical methods for noncovalent interactions are the highly correlated ab initio approaches [11], because they reliably describe the strength of all types of noncovalent bonding, as exemplified in references [12,13,14,15,16,17,18] (in the following, the strength of an intermolecular interaction will be expressed by the interaction energy, ΔE, in kJ/mol). Within the highly correlated approaches, the coupled cluster theory with singles, doubles, and iterative triples [CCSD(T)] is of a special significance. Namely, results of simpler methods for the ΔE prediction are frequently evaluated against the CCSD(T) ΔE value extrapolated to its complete basis set limit (CBS), as such value is considered to be sufficiently accurate for practically all applications. Since the canonical CCSD(T)/CBS computations are unfeasible for larger complexes (at present the limit is about 60 atoms [19]), reduced-scaling variants of the CCSD(T) method are used, which were most recently surveyed in reference [20]. Currently the domain-based local pair natural orbital (DLPNO) variant [21,22,23,24,25] of the CCSD(T) is the most important due to its particularly favorable scaling with the system size [26,27]. The DLPNO-CCSD(T)/CBS calculations can be employed to obtain the benchmark ΔE values for very large complexes (see references [28,29,30] and work cited therein, and also the related investigation of chemical reactivity [31]). Nevertheless, various simplified procedures for the DLPNO-CCSD(T)/CBS ΔE estimation are of interest. Examples include an application of the multiplicative CBS extrapolation protocol in reference [32]; using smaller basis sets to obtain the DLPNO-CCSD(T) correction term as described in reference [28]; and extrapolating energies computed with certain reductions in the DLPNO correlation space in reference [33]. These and other simplified procedures aim at reducing the computational cost of the underlying calculations while retaining the quality of a CBS-extrapolated result. The present investigation is also oriented toward this objective. Namely, a general goal of this work is to devise the DLPNO-CCSD(T) based prediction scheme for obtaining completely reliable ΔE values at a relatively low computational cost. In the initial step toward achieving this goal, a diverse testing set of 27 complexes is selected and its reference canonical CCSD(T)/CBS data are obtained together with a careful characterization of the intermolecular binding by means of the DFT-SAPT, as described in Section 2.1. In the next step, the basis set incompleteness error and settings that affect the quality of the DLPNO-CCSD(T) results are studied, and a robust procedure is described that is based on the focal-point analysis [34] of the ΔE data computed using two larger basis sets (see Section 2.2). This procedure is validated using the aforementioned testing set and also for systems from the renowned S22 collection [35]. In the subsequent step, which is detailed in Section 2.3, an even cheaper method is presented. It is based on the fitting of the benchmark values of absolute energies from the S22 dataset to their counterparts, computed using two smaller basis sets. The parameters thus obtained serve for an estimation of the ΔE values of complexes from the aforementioned testing set, which enables establishment of the accuracy limits of this computationally cheap approach. Since the fitting procedure is found to lead to quite reliable results, it is applied to large systems in the final step of this investigation (see Section 2.4 and Section 3). Specifically, for two polymorphic forms of poly(3-hydroxybutyrate) (PHB) that were previously characterized experimentally [36,37], dimeric models of PHB chains are considered. Moreover, three systems from the L7 dataset of large complexes [38] are examined: the parallel-displaced dimer of coronene (C2C2PD), the guanine trimer (GGG), and the tetramer consisting of two guanine–cytosine pairs (GCGC), as their ΔE values predicted by the CCSD(T) and the Quantum Monte Carlo (QMC) [39] approaches are a matter of the ongoing debate (see, in particular, references [40,41,42,43]).

## 2. Results

### 2.1. The Reference Interaction Energies

First the benchmark ΔE values had to be established for their use in a development of simpler predicting scheme(s). Thus, for a total of 47 complexes (the S22 dataset and 27 systems that are specified in Materials and Methods section), the canonical CCSD(T)/CBS interaction energies, $\Delta E_{\mathrm{CCSD}\left(T\right)}^{\mathrm{CBS}}$, were obtained. Throughout this work, the standard correlation correlation-consistent polarized-valence basis sets augmented with one set of diffuse basis functions were used (related double-zeta, triple-zeta, quadruple-zeta, and quintuple-zeta basis set is abbreviated as aDZ, aTZ, aQZ, and a5Z, respectively), and the counterpoise correction [44] was applied to reduce the basis set superposition error. In order to estimate a value of $\Delta E_{\mathrm{CCSD}\left(T\right)}^{\mathrm{CBS}}$, the focal-point method expressed by Equation (1) was applied to underlying energies (the basis set used to obtain the respective term is specified in the superscript).

(1) $$\Delta E_{\mathrm{CCSD}\left(T\right)}^{\mathrm{CBS}}=\Delta E_{\mathrm{HF}}^{a5Z}+\Delta E_{\mathrm{MP}2}^{a5Z}+\Delta E_{\mathrm{post}-\mathrm{MP}2}^{\mathrm{aTZ}}$$

The respective portions of the total interaction energy are the Hartree–Fock component, $\Delta E_{\mathrm{HF}}$, the second-order Møller–Plesset (MP2) correlation energy component, $\Delta E_{\mathrm{MP}2}$, and the correction for higher-order correlation energy contributions that is denoted as $\Delta E_{\mathrm{post}-\mathrm{MP}2}$ and approximated as a difference of the corresponding CCSD(T) and MP2 correlation energies (see the review [45] for discussion). The procedure from Equation (1) was carefully checked for the S22 dataset using the values from reference [46] (so called S22B set). Importantly, the fit of present $\Delta E_{\mathrm{CCSD}\left(T\right)}^{\mathrm{CBS}}$ results to their counterparts from the S22B collection is almost perfect (see Figure S1; the raw data are shown in Table S1). The highest absolute and relative differences between the two data sets are as low as 0.92 kJ/mol and 2.8%, respectively (they accordingly occur for the dimer of formic acid and for the benzene∙∙∙methane complex), while the mean absolute deviation is only 0.32 kJ/mol. Hence, the method expressed by Equation (1) was applied also to the 27 testing systems. All underlying absolute energies are provided in Supporting Information inside the Excel spreadsheet ‘energies1.xlsx’ for $\Delta E_{\mathrm{CCSD}\left(T\right)}^{\mathrm{CBS}}$ estimation using Equation (1).

For all complexes investigated in this work, which are listed in Table 1, the physical nature of intermolecular bonding was described by means of the SAPT-DFT calculations. It is thus important to ascertain the accuracy of the employed SAPT-DFT/CBS computational protocol, whose details are given in Section 4. This check was performed for a subset of 18 dimers. This assembly is called ‘Set3x6′, because it can be divided into three groups, with six complexes in each group, on the basis of the dispersion-to-polarization ratio [47]. Namely, the respective Set3x6 groups contain electrostatics-dominated, mixed, and dispersion-dominated dimers (see Table 1). For all three groups, an agreement between the SAPT-DFT/CBS and $\Delta E_{\mathrm{CCSD}\left(T\right)}^{\mathrm{CBS}}$ data is fairly good and uniform (see Figure S2 and Table S2). The biggest absolute and relative discrepancy between these two data sets is 1.75 kJ/mol and 13.7% exhibited by anisole∙∙∙CO_2_ and the acetylene dimer, respectively, and the root-mean-square deviation is 0.84 kJ/mol, which is much less than 2.05 kJ/mol reported for a similar comparison for the S22 dataset [45] (in that case, smaller basis sets were used). This indicates that all SAPT-DFT/CBS results presented in this work are fully reliable.

### 2.2. Comparing the Canonical and DLPNO-Based CCSD(T) Data

As already mentioned, for larger complexes, the canonical $\Delta E_{\mathrm{CCSD}\left(T\right)}^{\mathrm{CBS}}$ computations would be impractical, and a local electron-correlation scheme, such as the DLPNO method, would need to be applied. Using the DLPNO approximation, there are two ways of estimating the $\Delta E_{\mathrm{CCSD}\left(T\right)}^{\mathrm{CBS}}$ value. In the first approach, the DLPNO-CCSD(T) energies are obtained for a series of some correlation-consistent basis sets and extrapolated to the CBS limit. This approach may very quickly reach computational restrictions if applied to large systems. Nevertheless, it was used for the 49 dimers described in the preceding part, and its results are used for comparison purposes (see below). The second approach to the $\Delta E_{\mathrm{CCSD}\left(T\right)}^{\mathrm{CBS}}$ estimation applies a composite scheme that is analogous to the focal point analysis from Section 2.1. The present composite approach is expressed by Equation (2) (the right arrow indicates an extrapolation of the respective energy term to its CBS limit by applying the two-point formula from reference [53]). It is implemented in the Excel spreadsheet ‘energies2.xlsx’ (see Supporting Information), where all the underlying absolute energies can be found.

(2) $$\Delta E_{\mathrm{CCSD}\left(T\right)}^{\mathrm{CBS}}=\Delta E_{\mathrm{HF}}^{\mathrm{aQZ}}+\Delta E_{\mathrm{MP}2}^{\mathrm{aTZ}\to \mathrm{aQZ}}+\Delta E_{\mathrm{post}-\mathrm{MP}2}^{\mathrm{aTZ}\to \mathrm{aQZ}}$$

It should be noted that the $\Delta E_{\mathrm{post}-\mathrm{MP}2}$ term was taken as a difference of the pertinent DLPNO-CCSD(T) and DLPNO-MP2 [54] correlation energies. These energies were obtained with tight thresholds of the DLPNO approximation (see Section 4 for details). A contribution of triple excitations to the correlation energy was approximated by the non-iterative calculations, which are sometimes denoted as (T_0_), instead of using the iterative scheme that would provide results denoted as (T_1_) [24]. This choice was made on the basis of a significant increase of computational time of the DLPNO-CCSD(T_1_) calculations with respect to their DLPNO-CCSD(T_0_) counterparts, and by negligible differences between the two sets of results for dimers from the Set3x6 (see Figure S3 and Table S3).

**Figure 1:**
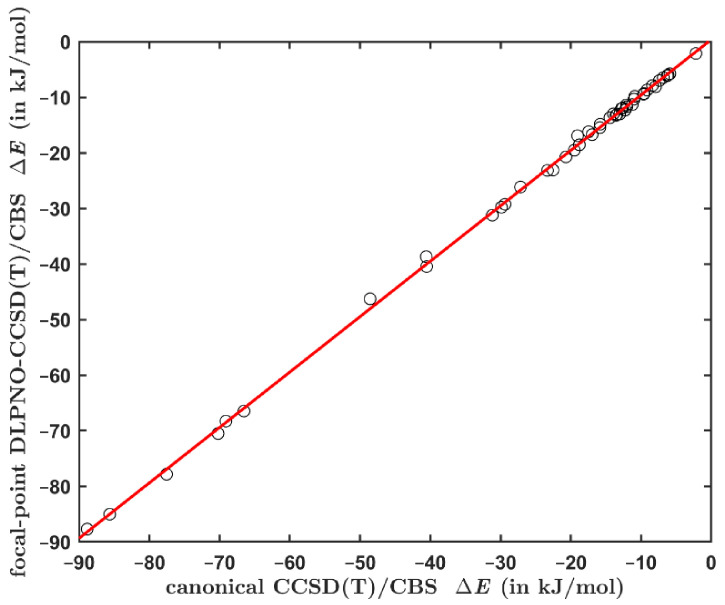
Comparison of the interaction energies computed for the set of 49 dimers by an application of protocols from Equations (1) and (2). The regression line is specified in the text and shown in red.

The $\Delta E_{\mathrm{CCSD}\left(T\right)}^{\mathrm{CBS}}$ data obtained using Equations (1) and (2) for the aforementioned set of 49 complexes span a large interval of values from ca. 2 to ca. 89 kJ/mol, and are listed in Table S4. Clearly, the DLPNO-based interaction energies closely match their canonical counterparts (see Figure 1). The maximum absolute difference between these data points is 2.29 kJ/mol exhibited by the stacked adenine∙∙∙thymine (AT) pair. It should be noted that there is also a small uncertainty in the canonical $\Delta E_{\mathrm{CCSD}\left(T\right)}^{\mathrm{CBS}}$ values (see a related discussion for the S22 set in reference [55]). The linear regression is {y} = 0.9971 × {x} + 0.3766 kJ/mol (shorthand notation is used with {y} for the DLPNO-based and {x} for the canonical $\Delta E_{\mathrm{CCSD}\left(T\right)}^{\mathrm{CBS}}$ data, respectively), with adjusted $R^{2}\;$= 0.9993 and the standard deviation of 0.59 kJ/mol. The maximum residual of this fit is 1.77 kJ/mol, and expectedly occurs for the stacked AT pair. However, such discrepancy amounts to only ca. 3.8% of the canonical $\Delta E_{\mathrm{CCSD}\left(T\right)}^{\mathrm{CBS}}$ value of ca. 48.6 kJ/mol obtained for this complex. The highest relative error is found for the stacked indole∙∙∙benzene dimer. In this case, the residual is 1.69 kJ/mol, which is ca. 8.9% of the canonical $\Delta E_{\mathrm{CCSD}\left(T\right)}^{\mathrm{CBS}}$ value of ca. 19.0 kJ/mol (see Table S4).

**Figure 2:**
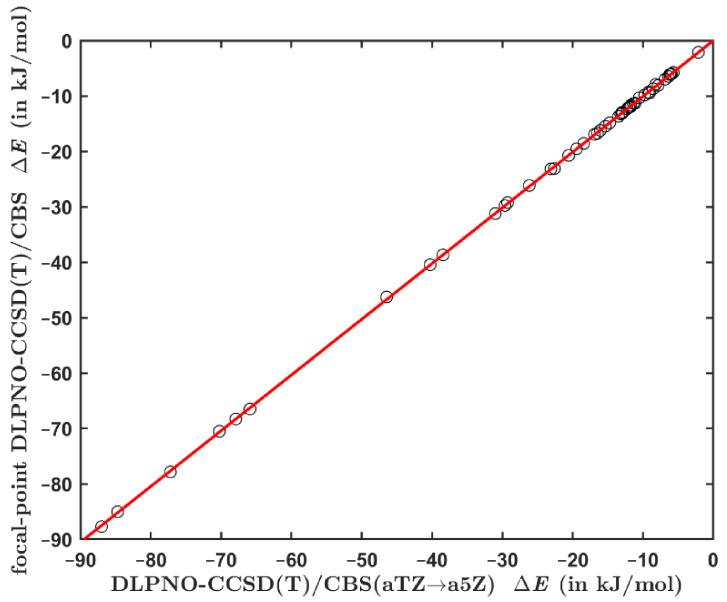
Comparison of the interaction energies computed for the set of 49 dimers by an application of the procedure from reference [56] and the protocol from Equation (2). The regression line is shown in red.

The interaction energies computed using the focal point method from Equation (2) were also checked against yet another set of results. Namely, the total DLPNO-CCSD(T) energies were calculated while employing the aTZ; aQZ; a5Z series of basis sets, and extrapolated to their CBS limit using the mixed Gaussian/exponential form from reference [54]. The underlying energies are provided in the Excel spreadsheet ‘energies3.xlxs’ together with an analytical solution to the set of three equations from reference [56], which is used to compute the pertinent $\Delta E_{\mathrm{CCSD}\left(T\right)}^{\mathrm{CBS}}$ value (see Supporting Information). Figure 2 presents an excellent agreement between the two sets of DLPNO-based CCSD(T)/CBS interaction energies (their values are collected in Table S4). This result shows that there is only a negligible basis set incompleteness error in the $\Delta E_{\mathrm{CCSD}\left(T\right)}^{\mathrm{CBS}}$ results obtained by the focal point method expressed by Equation (2). As a consequence, the DLPNO-CCSD(T)/a5Z calculations should not be needed for an accurate estimation of the $\Delta E_{\mathrm{CCSD}\left(T\right)}^{\mathrm{CBS}}$ data.

### 2.3. The Fittings Scheme for Smaller Basis Sets

While the composite procedure from the previous part (Equation (2)) is clearly successful in reliably predicting the interaction energies, it also requires results obtained using the aQZ basis set, which would be impractical for very large systems. Hence, an attempt was made to devise some less costly computational protocol. It is stressed that a direct application of Equation (2) together with smaller basis sets (aDZ and aTZ, for instance) cannot be expected to lead to an accurate estimate of the $\Delta E_{\mathrm{CCSD}\left(T\right)}^{\mathrm{CBS}}$ [57]. Instead, all three terms on the right-hand side of Equation (2) need to be extrapolated to their CBS limits, which in the following will be designated $\Delta E_{\mathrm{HF}}^{\mathrm{CBS}*}$, $\Delta E_{\mathrm{MP}2}^{\mathrm{CBS}*}$, and $\Delta E_{\mathrm{post}-\mathrm{MP}2}^{\mathrm{CBS}*}$, in order to assume their sum, $\Delta E_{\mathrm{CCSD}\left(T\right)}^{\mathrm{CBS}*}$, to be close to a true value of the $\Delta E_{\mathrm{CCSD}\left(T\right)}^{\mathrm{CBS}}$ term [41]. A particular attention has to be paid to the choice of respective extrapolation schemes for $\Delta E_{\mathrm{HF}}^{\mathrm{CBS}*}$, $\Delta E_{\mathrm{MP}2}^{\mathrm{CBS}*}$, and $\Delta E_{\mathrm{post}-\mathrm{MP}2}^{\mathrm{CBS}*}$ contributions, as each of the underlying energy components converges differently with increasing the basis set size and quality (see the most recent study [58] and references cited therein). For an extrapolation of the HF and correlation energies, in frequent use are the exponents tabulated in reference [59] that were obtained by fitting the absolute energies of 21 small molecules computed using a number of pairs of various basis sets. The pertinent exponents from Table 3 of reference [59] were applied to extrapolate the energies computed using the (aDZ, aTZ) basis sets for systems investigated here, but this led to inaccurate ΔE values (not shown) in some cases. Hence, all relevant energies for S22 dataset were obtained (58 values for each component of the total energy and basis set, because of the symmetry in 7 out of 22 clusters) and fitted as follows. For the HF energy, the coefficient *α* minimizes (in the least-squares sense) differences between $E_{\mathrm{HF}}^{a5Z}$ data and the functional form given by Equation (3):

(3) $$E_{\mathrm{HF}}^{\mathrm{fit}}\left(\alpha ;\;E_{\mathrm{HF}}^{\mathrm{aDZ}},E_{\mathrm{HF}}^{\mathrm{aTZ}}\right)=\frac{\exp\left(\alpha \sqrt{3}\right)E_{\mathrm{HF}}^{\mathrm{aDZ}}-\exp\left(\alpha \sqrt{2}\right)E_{\mathrm{HF}}^{\mathrm{aTZ}}}{\exp\left(\alpha \sqrt{3}\right)-\exp\left(\alpha \sqrt{2}\right)}$$

The fit gives *α* = −4.473 (rounding to four digits is performed on the basis of an estimated covariance that is not shown). The correlation energies were treated in an analogous way. Specifically, an optimal value of the coefficient *β* for the MP2 correlation energy contribution was obtained by minimizing differences between $E_{\mathrm{MP}2}^{a5Z}$ data and their $E_{\mathrm{MP}2}^{\mathrm{fit}}$ counterparts from Equation (4):

(4) $$E_{\mathrm{MP}2}^{\mathrm{fit}}\left(\beta ;\;E_{\mathrm{MP}2}^{\mathrm{aDZ}},E_{\mathrm{MP}2}^{\mathrm{aTZ}}\right)=\frac{E_{\mathrm{MP}2}^{\mathrm{aDZ}}2^{\beta }-E_{\mathrm{MP}2}^{\mathrm{aTZ}}3^{\beta }}{2^{\beta }-3^{\beta }}$$

This value is *β* = 2.796. As for the post-MP2 correlation energy term, numerical tests revealed that yet another coefficient would be needed, which is denoted as *γ* and minimizes differences between $\Delta E_{\mathrm{post}-\mathrm{MP}2}^{\mathrm{aTZ}}$ data and the functional form expressed by Equation (5):

(5) $$E_{\mathrm{post}-\mathrm{MP}2}^{\mathrm{fit}}\left(\gamma ;\;E_{\mathrm{post}-\mathrm{MP}2}^{\mathrm{aDZ}},E_{\mathrm{post}-\mathrm{MP}2}^{\mathrm{aTZ}},\beta \right)=\frac{2^{\gamma }E_{\mathrm{CCSD}\left(T\right)}^{\mathrm{aDZ}}-3^{\gamma }E_{\mathrm{CCSD}\left(T\right)}^{\mathrm{aTZ}}}{2^{\gamma }-3^{\gamma }}-\frac{2^{\beta }E_{\mathrm{MP}2}^{\mathrm{aDZ}}-3^{\beta }E_{\mathrm{MP}2}^{\mathrm{aTZ}}}{2^{\beta }-3^{\beta }}$$

**Figure 3:**
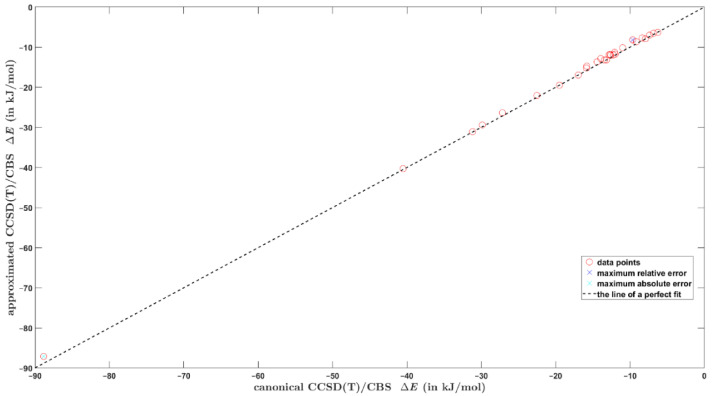
Comparison of the interaction energies computed for the testing set of 27 dimers by two methods that are discussed in the text. The raw data are available from Table S5.

An optimal value of this coefficient is found to be *γ* = 2.741 for the parameter β kept constant at β = 2.796 (see above). The data sets that were actually used for fitting are included in Supporting Information. For the testing set of aforementioned 27 dimers, the $\Delta E_{\mathrm{CCSD}\left(T\right)}^{\mathrm{CBS}*}$ values were obtained through Equations (3)–(5) and compared to their $\Delta E_{\mathrm{CCSD}\left(T\right)}^{\mathrm{CBS}}$ counterparts, which are described in Section 2.1. The data are collected in Table S4 and graphically presented in Figure 3, and illustrate a good performance of present fitting scheme for cost-effective estimation of the ΔE. In Figure 3 the highest absolute and relative differences are marked. They amount to 1.72 kJ/mol and 17.9%, respectively, and are accordingly exhibited by the cyclopropenium cation∙∙∙anthracene complex with large interaction energy of almost –90 kJ/mol, and by the highly challenging configuration of furan∙∙∙toluene dimer (see Discussion). The linear regression model is $\Delta E_{\mathrm{CCSD}\left(T\right)}^{\mathrm{CBS}*}\;$= 0.9987 × $\Delta E_{\mathrm{CCSD}\left(T\right)}^{\mathrm{CBS}}\;$+ 0.3789 kJ/mol with adjusted $R^{2}$ = 0.9998 and the standard deviation of 0.45 kJ/mol. However, it should be mentioned that if the same testing set is treated using the procedure from Equation (2) that employs also the aQZ data, a significantly better agreement between the two sets of $\Delta E_{\mathrm{CCSD}\left(T\right)}^{\mathrm{CBS}}$ values (namely, those given by Equations (1) and (2)) is obtained. Specifically, the highest absolute and relative differences become as low as 0.71 kJ/mol and 4.2%, respectively (they occur for the same systems as in the case of an application of the computationally cheaper procedure). The procedure expressed by Equation (2) should thus be used whenever permitted by the size of an investigated system.

### 2.4. Testing Large Systems

**Figure 4:**
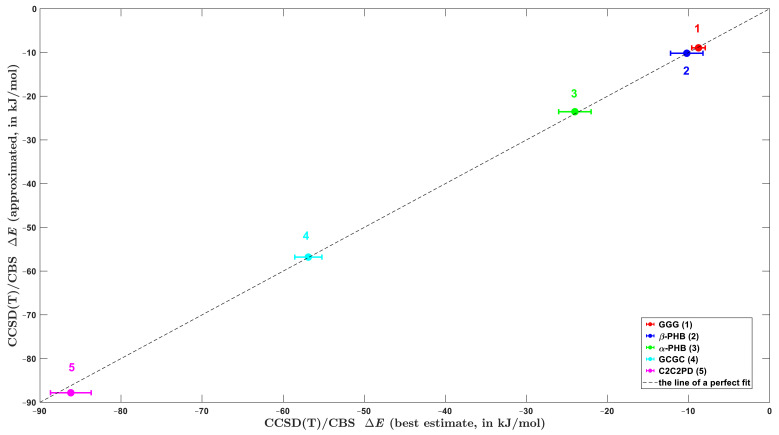
Comparison of the interactions energies of five large complexes that are discussed in the text. The error bars that are plotted for systems number 1, 4, and 5 are given in Table 1. For systems number 2 and 3, the error bars of ±2 kJ/mol are plotted.

The procedure from Section 2.3 was of course devised with applications to extended systems in mind. Thus, it needs to be validated by checking its accuracy also for intermolecular complexes that are significantly larger than those listed in Table S4, because the local approximation error growths with the system size (see reference [60] and work cited therein), and this problem might be exacerbated by using relatively small (aDZ, aTZ) basis sets. Since the canonical $\Delta E_{\mathrm{CCSD}\left(T\right)}^{\mathrm{CBS}}$ data are not available, the SAPT-DFT/CBS calculations were applied to obtain reference values of the interaction energy for models of two polymer chains (see Materials and Methods). Additionally, the DLPNO-based focal point method from Section 2.2 was used for comparison purposes. The same methods were also applied to three challenging systems from the L7 dataset. Namely, C2C2PD and GCGC were chosen due to known differences in their ΔE values as obtained from the CCSD(T) and QMC computations [43], while GGG was included because of an exceedingly high amount of the dispersion contribution to the stabilization of this complex, leading to the dispersion-to-polarization ratio of about six (see Table 1). Table 2 summarizes results provided by the DLPNO-based methods together with their SAPT-DFT counterparts (the benchmark data for C2C2PD, GCGC and GGG from reference [43] are shown in Table 1 together with their error bars). It is evident that there are no apparent outliers among these results. Figure 4 compares the best estimates of the ΔE values to their counterparts obtained using the cost-effective procedure expressed by Equations (3)–(5). Namely, results from reference [43] are employed in the ordinate for C2C2PD, GCGC, and GGG. For the models of PHB polymorphs, interaction energies obtained by an application of Equation (2) are used together with an uncertainty estimate of ±2 kJ/mol, which is discussed in the subsequent part.

## 3. Discussion

**Figure 5:**
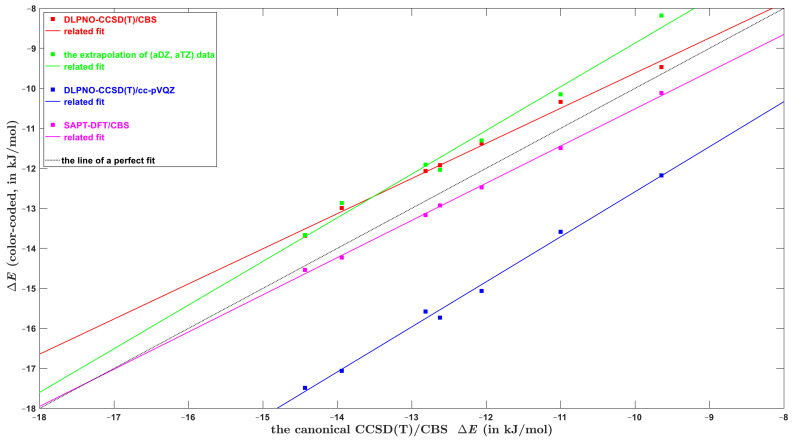
Comparison of the interaction energies of seven configurations of furan∙∙∙toluene dimer.

The DLPNO-based methods from Section 2.2 and Section 2.3 showed a good performance in predicting absolute values of the CBS-extrapolated binding energies for a variety of molecular clusters. In particular, if the (aTZ, aQZ) data were used in approach expressed by Equation (2), the ensuing $\Delta E_{\mathrm{CCSD}\left(T\right)}^{\mathrm{CBS}}$ should lie within about 2.0 kJ/mol of a true absolute value of the interaction energy. Thus, they would be expected to lead to the ΔE result of at least “bronze standard” quality [61]. Moreover, an application of the refitted exponents (Equations (3)–(5)) to the (aDZ, aTZ) data was found to work remarkably well for larger systems (see Table 2). Nevertheless, in areas of, for instance, conformational analysis (see the most recent investigation [62] and references cited therein), computer-assisted rotational spectroscopy [63], developing new structural descriptors [48], or for anticipated applications in modeling of polymers, the relative energies of various configurations of an investigated system also need to be obtained with a high accuracy. A stringent test was performed here using seven stacked orientations of the furan∙∙∙toluene dimer (they are numbered as they consecutively appear in the supporting information to reference [48]). It can be verified by a visual inspection that the complexes are fairly different from each other. However, their interaction energies span a narrow interval of ca. 4 kJ/mol (see Figure 5; raw values as provided by the respective methods are available from Table S6). As follows from the SAPT energy decomposition (see Table 1), the dispersion interaction completely dominates the intermolecular bonding in these clusters. It should be noted that in one case (namely, for the configuration #7) the dispersion-to-polarization ratio is as high as 4.5. This is the structure with highest differences between the DLPNO-based and canonical $\Delta E_{\mathrm{CCSD}\left(T\right)}^{\mathrm{CBS}}$ results described in Section 2.3. Four computational approaches were applied to these furan∙∙∙toluene dimers in addition to the benchmarking canonical $\Delta E_{\mathrm{CCSD}\left(T\right)}^{\mathrm{CBS}}$ calculations, and key statistical parameters describing the level of agreement between the relevant datasets are shown in Table S7. The $\Delta E_{\mathrm{CCSD}\left(T\right)}^{\mathrm{CBS}}$ results that were obtained from the focal-point analysis of the (aTZ, aQZ) data are designated ‘DLPNO-CCSD(T)/CBS’ in Figure 5. They are accurate in both absolute and relative terms, as are the SAPT-DFT/CBS values for this challenging set of complexes. An inspection of Figure 5 reveals that the ΔE results that were not extrapolated to the CBS limit, namely, the DLPNO-CCSD(T)/cc-pVQZ data from reference [48], are incorrectly ordered. Moreover, they are heavily shifted with respect to the canonical $\Delta E_{\mathrm{CCSD}\left(T\right)}^{\mathrm{CBS}}$ values (on average by 2.88 kJ/mol), but this shift was already discussed in reference [48]. The ordering of interaction energies estimated through Equations (3)–(5) is also incorrect (see Figure 5), and a significant underestimation of the ΔE occurs for the aforementioned configuration #7. These results for the furan∙∙∙toluene dimers indicate that the relative accuracy of the scheme expressed by Equation (2) should be around a half of kJ/mol. Consequently, this approach is well-suited for checking results of less demanding methods, for example, variants of DFT that were tailored for noncovalent interactions [64]. The computationally much cheaper procedure that applies Equations (3)–(5) can be expected to have the relative accuracy of about one kJ/mol. This level of accuracy is unlikely to be limiting in applications to modeling large systems, though, as for them there would probably be more significant uncertainties due to the geometry and media effects [65]. Nevertheless, further testing of both procedures (those expressed by Equation (2) and by Equations (3)–(5)) is desirable.

## 4. Materials and Methods

The following 11 dimers were considered in their MP2/aTZ geometry from reference [66]: aniline∙∙∙methane, anisole∙∙∙methane, 1-naphthol∙∙∙methane, 1-naphthol∙∙∙CO, 1-naphthol∙∙∙CO_2_, anisole∙∙∙anisole, anisole∙∙∙ammonia, 1-naphtol∙∙∙acetylene, HCl∙∙∙HCl, benzene∙∙∙water, 1-naphtol∙∙∙ammonia, and HCl∙∙∙water. The MP2/aTZ geometries, which are provided in ‘geometries.tar’ file in Supporting Information, were obtained for the following 7 dimers: anisole∙∙∙CO_2_ (the optimization started from a structure analogous to the one showed in Figure 1 of reference [67]), acetylene∙∙∙acetylene in the T-shaped configuration (see reference [68]), HCN∙∙∙HF (see reference [50]), NCH∙∙∙FH (see reference [50]), HCN∙∙∙HCN (see reference [49]), and 1-naphthol∙∙∙water (the optimization started from a structure featuring a classical hydrogen bond between the hydroxyl oxygen of 1-naphtol and one of the protons of water). These 18 structures form the aforementioned ‘Set3x6′ testing suite. Each structure optimized at the MP2/aTZ level was verified to be a minimum of the potential energy surface by inspecting the predicted harmonic vibrational frequencies. Pertinent calculations were performed using the Gaussian 16, revision C.01 suite of codes [69] with default settings.

The dimeric models of the α and β polymorphs of PHB were prepared using coordinates from references [36,37], respectively. Their structures are included in ‘structures.zip’ file in Supporting Information.

The configurations 1–7 of the furan∙∙∙toluene dimer were taken from ‘ja9b00936_si_002.xyz’ file of supporting materials to reference [48]. Coordinates of the S22 dataset, the stacked pyridine∙∙∙pyridine, C2C2PD, GCGC, and GGG were downloaded from the BEGDB website [70]. Coordinates of the cyclopropenium∙∙∙anthracene dimer were taken from supporting materials to reference [16].

The SAPT-DFT, canonical CCSD(T) and MP2 calculations were carried out in Molpro 2021.2 [71]. The same procedures and notation as in our most recent work [66] were used for the SAPT-DFT analysis.

The MP2/a5Z energies for an application of Equation (1) were obtained in the resolution-of-the-identity integral approximation [72,73] while using the relevant auxiliary basis sets [74]. The program package Turbomole, version 7.1 [75] was used for these and for related HF/a5Z calculations.

The DLPNO-based computations were carried out in ORCA 5.0 [76]. The underlying HF calculations used ‘VeryTightSCF’ accuracy settings. The default ‘augmented Hessian Foster–Boys’ localization scheme was adopted. The truncation of the electron-correlation space was performed by applying the ‘TightPNO’ set of parameters.

The Levenberg–Marquardt algorithm as implemented in ‘lsqcurvefit’ function of MATLAB^®^ Optimization Toolbox™ was used to perform the nonlinear fitting of models expressed by Equations (3)–(5). The values of α, β, γ parameters thus found (see Equations (3)–(5)) were checked using ‘e04fcf’ routine of the NAG^®^ Fortran Library; the respective data files are included in Supporting Information.

## Figures and Tables

**Table 1:** The DFT-SAPT analysis of intermolecular clusters considered in this work (all values are in kJ/mol, and “1-Nap” stands for 1-naphtol). Parentheses are used in cases when the literature data contain error estimates.

Type of System	Description	Components of the DFT-SAPT Energy					Best Estimate of ΔE
		$\;E_{pol}$	$E_{exch}$	$E_{ind}$	$E_{disp}$	$E_{total}$	
dispersion-dominated complex from Set3x6	aniline:methane	–6.5	18.9	–1.9	–17.3	–6.8	–6.84 ^a^
	anisole:methane	–7.1	19.9	–1.6	–18.5	–7.3	–7.39 ^a^
	1-Nap:methane	–9.0	25.3	–1.8	–23.4	–8.9	–9.14 ^a^
	1-Nap:CO	–9.4	24.0	–3.4	–20.2	–9.0	–8.37 ^a^
	1-Nap:CO_2_	–13.1	28.6	–3.2	–24.6	–12.3	–12.68 ^a^
	anisole:anisole	–32.8	72.8	–8.6	–57.6	–26.1	–27.16 ^a^
mixed-interactions complex from Set3x6	anisole:ammonia	–16.1	24.3	–3.7	–15.7	–11.2	–12.00 ^a^
	1-Nap:ethyne	–25.1	35.0	–10.1	–17.4	–17.5	–16.96 ^a^
	HCl:HCl	–11.3	17.6	–6.3	–9.2	–9.2	–7.94 ^a^
	benzene:water	–13.5	19.2	–5.1	–14.7	–14.0	–13.43 ^a^
	anisole:CO_2_	–20.5	28.6	–3.5	–18.8	–14.1	–15.86 ^a^
	ethyne:ethyne	–9.0	11.6	–2.9	–6.9	–7.3	–6.26 ^a^
electrostatics-dominated complex from Set3x6	1-Nap:ammonia	–70.0	80.6	–28.7	–22.8	–40.9	–40.52 ^a^
	HCl:water	–41.5	50.7	–17.6	–14.3	–22.8	–22.47 ^b^
	HCN:HF	–42.6	42.9	–18.5	–11.8	–30.1	–31.09 ^c^
	NCH:FH	–15.4	11.5	–3.4	–5.2	–12.4	–12.34 ^c^
	HCN:HCN	–25.2	20.3	–6.8	–7.8	–19.5	–19.83 ^d^
	1-Nap:water	–46.2	50.0	–15.8	–16.6	–28.6	–29.86 ^a^
furan:toluene stacked complex from reference [48]	configuration #1	–9.0	26.5	–2.9	–29.2	–14.5	–14.43
	configuration #2	–8.9	27.6	–3.1	–29.8	–14.2	–13.94
	configuration #3	–7.3	26.5	–2.8	–29.3	–12.9	–12.62
	configuration #4	–7.7	25.9	–3.1	–28.3	–13.2	–12.82
	configuration #5	–7.6	25.6	–2.8	–27.8	–12.5	–12.06
	configuration #6	–6.7	23.7	–2.6	–25.9	–11.5	–11.00
	configuration #7	–5.3	20.8	–2.2	–23.5	–10.1	–9.65
miscellaneous	anthracene: cyclopropenium	–58.5	81.8	–60.3	–48.3	–85.3	(–89.96 ±0.84) ^e^
	pyridine:pyridine	–12.0	28.2	–3.3	–29.8	–16.8	–15.82 ^a^
large	α-PHB model	–14.7	17.5	–3.8	–25.1	–26.1	–24.03 ^f^
	β-PHB model	–8.1	29.1	–4.0	–28.4	–11.4	–10.23 ^f^
	C2C2PD	–37.1 –30.8 ^h^	114.3 107.2 ^h^	–12.8 –10.7 ^h^	–147.1 –154.3 ^h^	–82.7 –88.6 ^h^	(–87.82 ±2.51) ^g^
	GCGC	–37.4 –34.6 ^h^	104.1 97.5 ^h^	–9.5 –8.6 ^h^	–111.9 –115.1 ^h^	–54.7 –60.9 ^h^	(–56.90 ±1.67) ^g^
	GGG	11.3 12.0 ^h^	27.8 25.7 ^h^	–6.2 –5.6 ^h^	–39.9 –41.0 ^h^	–6.9 –8.9 ^h^	(–8.79 ±0.84) ^g^

^a^ CCSD(T)/CBS value obtained using Equation (1) in this work; ^b^ obtained as described at page 7109 of reference [49]; ^c^ obtained as described at page 3 of reference [50]; ^d^ obtained as described in Table 8 of reference [51]; ^e^ obtained as described in Table 1 of reference [16]; ^f^ DLPNO-CCSD(T)/CBS value obtained using Equation (2) in this work; ^g^ obtained as described in Table 1 of reference [43]; ^h^ obtained as described in the supporting information to reference [52].

**Table 2:** The negative of interaction energies (in kJ/mol) extrapolated to the complete basis set limit for large complexes.

Method	α-PHB	β-PHB	GGG	GCGC	C2C2PD
extrapolations using Equations (3)–(5)	23.56	10.18	8.95	56.79	87.81
the focal point analysis using Equation (2)	24.03	10.23	8.87	56.44	86.19
SAPT-DFT	26.14	11.43	6.88	54.69	82.68

## Data Availability

The data presented in this study are available in the article and in the Supplementary Materials.

## Supplementary Materials

The following supporting information can be downloaded at: https://www.mdpi.com/article/10.3390/ijms232415773/s1.
