# Supplementary material for: A Cost Effective Scheme for the Highly Accurate Description of Intermolecular Binding in Large Complexes
Source: Int J Mol Sci. 2022 Dec 12;23(24):15773. doi: 10.3390/ijms232415773 (PMC9780852; doi:10.3390/ijms232415773)
Supplement: Supplementary file 1 [file ijms-23-15773-s001.zip › SI.pdf]

Supporting Materials to "A cost effective scheme for the highly accurate description of intermolecular binding in large complexes" by Czernek, Brus and Czerneková (*IJMS* **2022**)

Contents

Figure S1: graphical presentation of the CCSD(T)/CBS interaction energies for the S22 set obtained from the S22B collection and in this work …………………………………. page SI2

Table S1: data for Figure S1 ……..………………………………………………….. page SI3

Figure S2: SAPT-DFT/CBS energies plotted against canonical CCSD(T)/CBS interaction energies for the Set3x6 ……………………………………………………………….. page SI4

Table S2: data for Figure S2 ………………………………………………………… page SI5

Figure S3: DLPNO-CCSD(T_0)/CBS interaction energies plotted against their DLPNO-CCSD(T_1)/CBS counterparts for the Set3x6 …………………………….................... page SI6

Table S3: data for Figure S3 ………………………………………………………… page SI7

Table S4: data for Figures 1 and 2 of the main text ……………………..… pages SI8 and SI9

Table S5: data for Figure 3 of the main text ……………………………………….. page SI10

Table S6: data for Figure 5 of the main text ……………………………………….. page SI11

Table S7: linear regression data for the furan···toluene dimer ……………………… page SI11

Figure S1. Graphical presentation of the CCSD(T)/CBS interaction energies for the S22 set obtained from the S22B collection (ref. **[44]**) and in this work. The linear regression is $\{y\} = 0.9902 \times \{x\} - 0.0015$, has the adjusted $R^2 = 0.9999$ and the standard deviation of 0.28 kJ/mol.

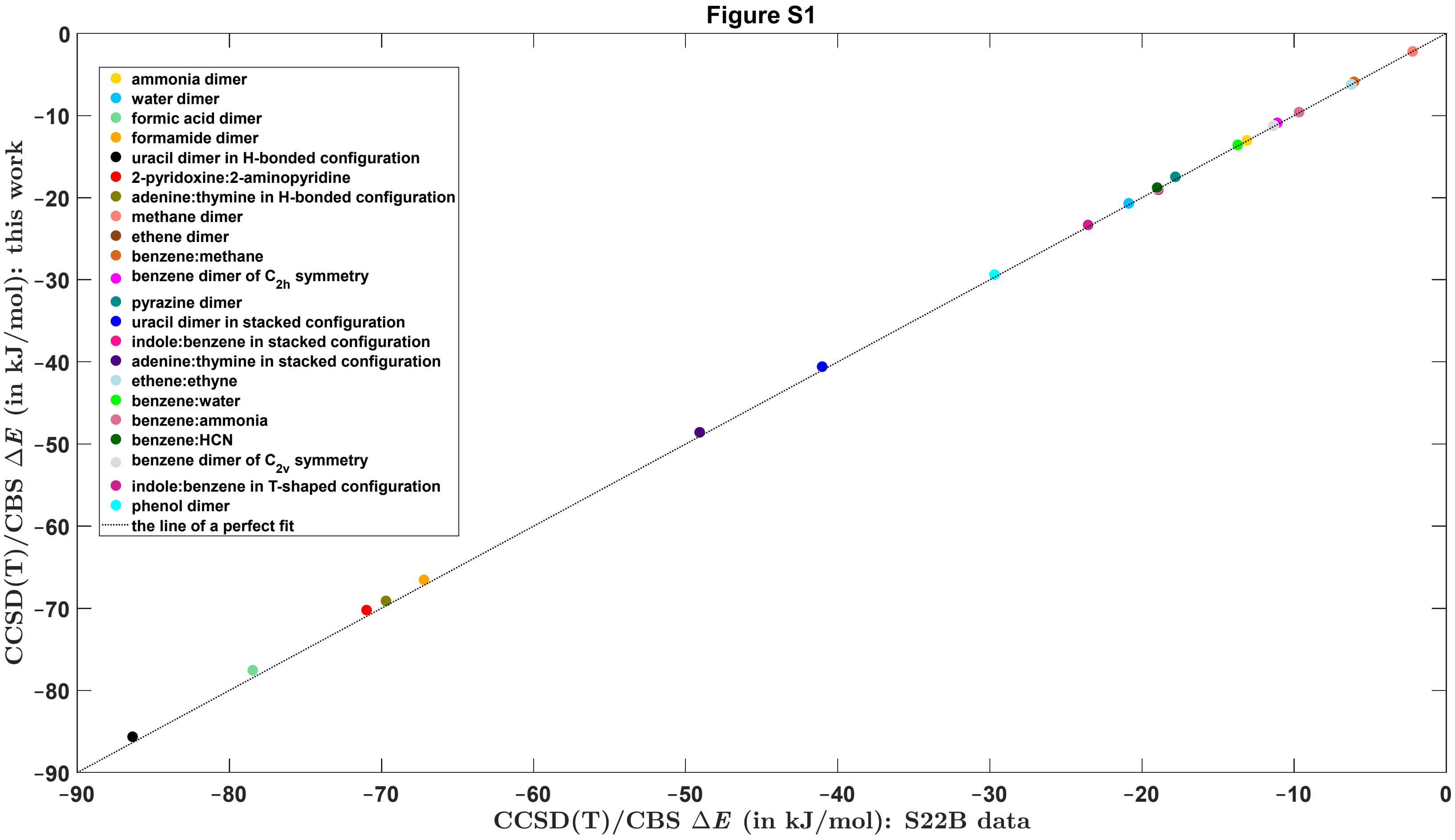

Table S1. CCSD(T)/CBS interaction energies, ΔE, for the S22 set obtained from the S22B collection (ref. **[44]**) and in this work. These values are plotted in Figure S1.

system		$-\Delta E$ (in kJ/mol)	
#	description	from ref. **[44]**	this work
S01	ammonia dimer	13.1085	13.0305
S02	water dimer	20.8740	20.6922
S03	formic acid dimer	78.4626	77.5433
S04	formamide dimer	67.2034	66.5291
S05	uracil dimer in H-bonded configuration	86.3619	85.6371
S06	2-pyridoxine:2-aminopyridine	70.9774	70.2161
S07	adenine:thymine in H-bonded configuration	69.7054	69.1001
S08	methane dimer	2.2050	2.1896
S09	ethene dimer	6.1588	6.1332
S10	benzene:methane	6.0584	5.8910
S11	benzene dimer of C_{2h} symmetry	11.1043	10.8905
S12	pyrazine dimer	17.8029	17.4600
S13	uracil dimer in stacked configuration	41.0241	40.5791
S14	indole:benzene in stacked configuration	18.9284	19.0416
S15	adenine:thymine in stacked configuration	49.0783	48.5693
S16	ethene:ethyne	6.2593	6.2466
S17	benzene:water	13.7026	13.5639
S18	benzene:ammonia	9.6734	9.5923
S19	benzene:HCN	18.9995	18.7783
S20	benzene dimer of C_{2v} symmetry	11.3679	11.2461
S21	indole:benzene in T-shaped configuration	23.5434	23.3231
S22	phenol dimer	29.6938	29.3670

Figure S2. Graphical presentation of the canonical CCSD(T)/CBS interaction energies, ΔE, and the SAPT-DFT/CBS energies, E, obtained for the Set3x6. The linear fit (shown as the blue line) is $E = 0.9632 \times \Delta E - 0.5388$ kJ/mol with the adjusted $R^2 = 0.9933$ and the standard deviation of 0.84 kJ/mol.

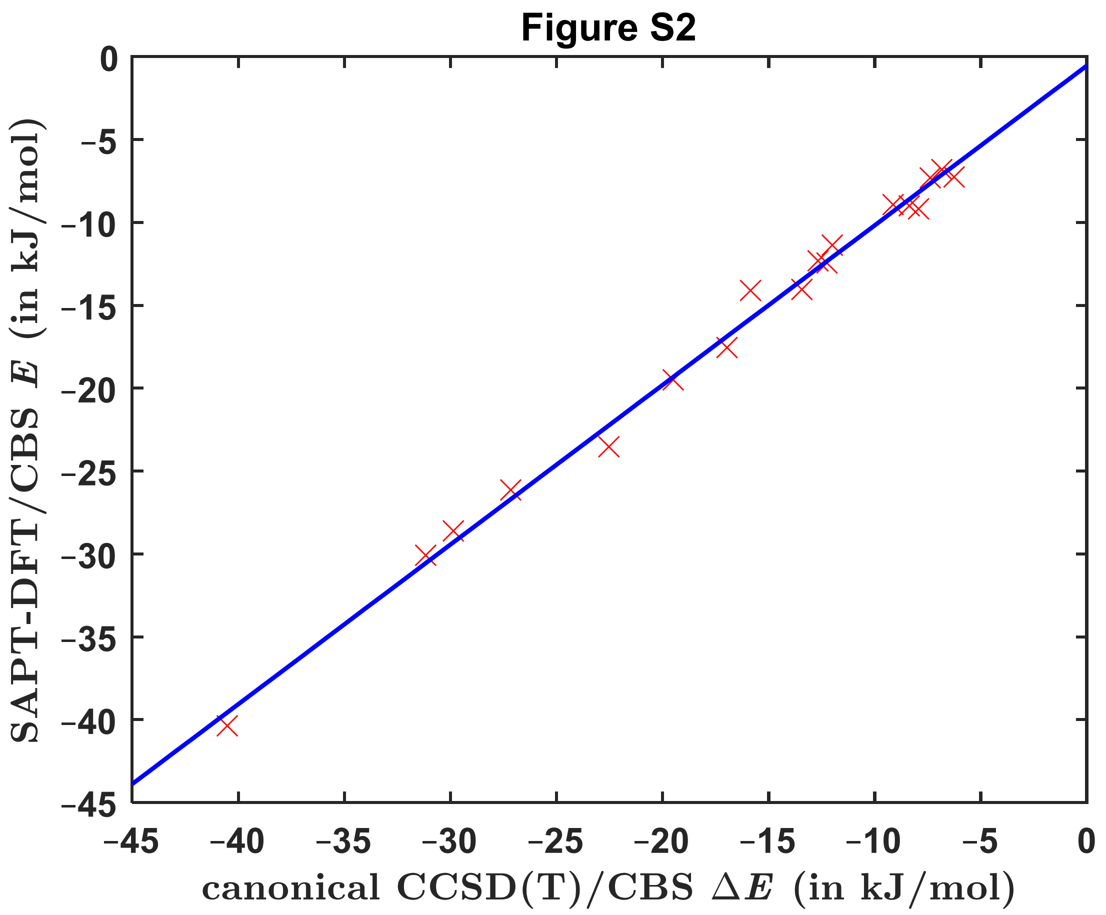

Table S2. The canonical CCSD(T)/CBS interaction energies, ΔE, and the SAPT-DFT/CBS energies, E, obtained for the Set3x6. These values are plotted in Figure S2.

dimer	CCSD(T)/CBS $-\Delta E$ (in kJ/mol)	SAPT-DFT/CBS $-E$ (in kJ/mol)
aniline:methane	6.8409	6.8076
anisole:methane	7.3866	7.3111
1-naphtol:methane	9.1377	8.9242
1-naphtol:CO	8.3737	8.9992
1-naphtol:CO_2	12.6781	12.3131
anisole:anisole	27.1599	26.1397
anisole:ammonia	12.0031	11.3728
1-naphtol:ethyne	16.9643	17.5480
HCl:HCl	7.9397	9.1796
benzene:water	13.4345	14.0423
anisole:CO_2	15.8595	14.1078
ethyne:ethyne	6.2616	7.2559
1-naphtol:ammonia	40.5199	40.3692
HCl:water	22.5321	23.5324
HCN:HF	31.1699	30.0755
NCH:FH	12.2561	12.4459
HCN:HCN	19.5007	19.4982
1-naphtol:water	29.8641	28.6100

Figure S3. Graphical presentation of the DLPNO-CCSD(T_1)/CBS ('iterative DLPNO-CCSD(T)/CBS' in the abscissa) interaction energies and their DLPNO-CCSD(T_0)/CBS ('noniterative DLPNO-CCSD(T)/CBS' in the ordinate) counterparts for the Set3x6. The linear regression is $\{y\} = 0.9989 \times \{x\} + 0.1451$, has the adjusted $R^2 = 0.9998$ and the standard deviation of 0.14 kJ/mol.

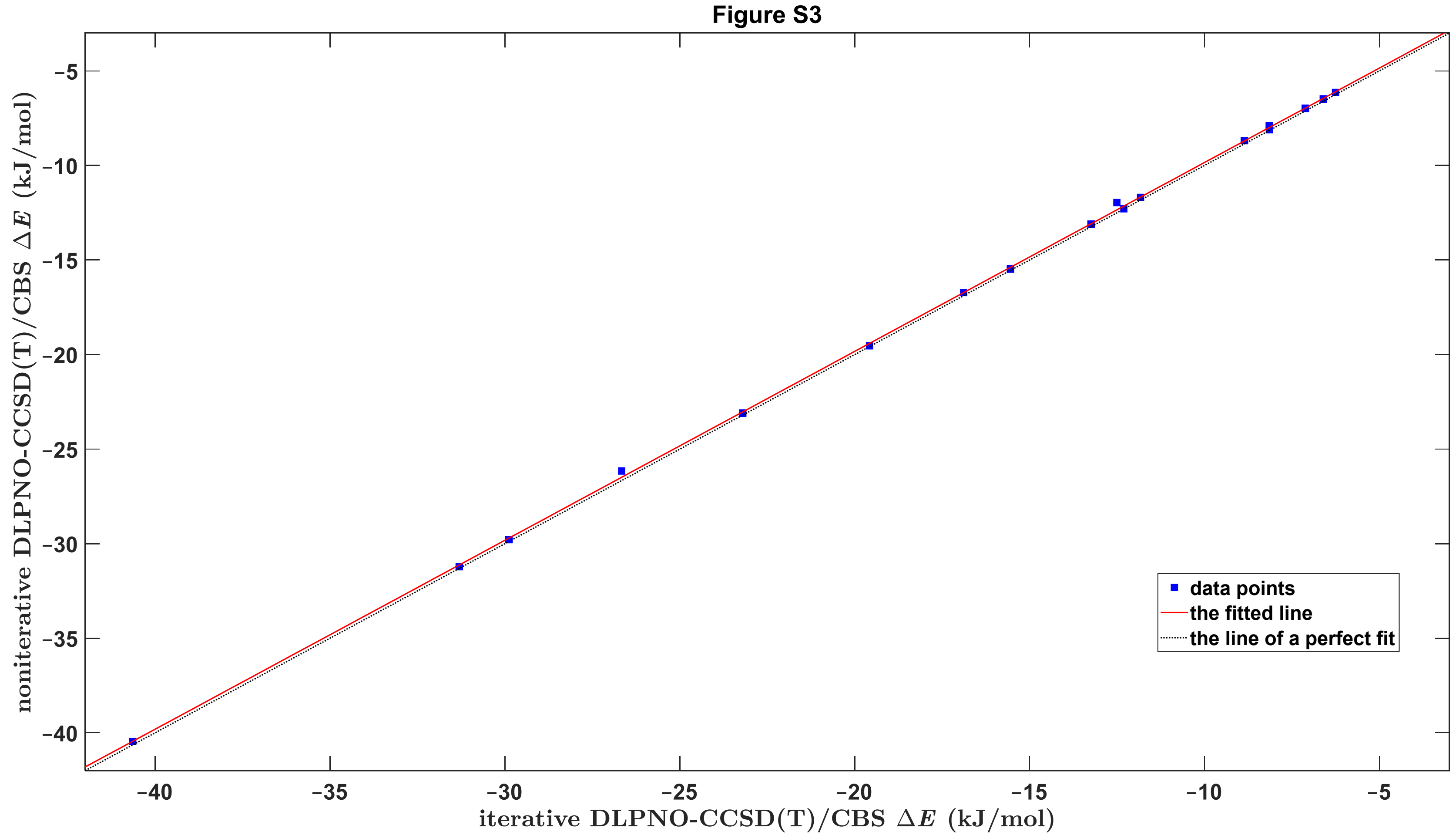

Table S3. The negative of the DLPNO-CCSD(T_1)/CBS (‘iterative DLPNO-CCSD(T)/CBS’) and the DLPNO-CCSD(T_0)/CBS (‘noniterative DLPNO-CCSD(T)/CBS’) interaction energy data for the Set3x6. These values are plotted in Figure S3.

dimer	iterative DLPNO-CCSD(T)/CBS (in kJ/mol)	noniterative DLPNO-CCSD(T)/CBS (in kJ/mol)
aniline:methane	6.8409	6.4802
anisole:methane	7.3866	6.9824
1-naphtol:methane	9.1377	8.6859
1-naphtol:CO	8.3737	7.8983
1-naphtol:CO_2	12.6781	11.9629
anisole:anisole	27.1599	26.1590
anisole:ammonia	12.0031	11.6934
1-naphtol:ethyne	16.9643	16.7201
HCl:HCl	7.9397	8.1103
benzene:water	13.4345	13.1087
anisole:CO_2	15.8595	15.4662
ethyne:ethyne	6.2616	6.1367
1-naphtol:ammonia	40.5199	40.4598
HCl:water	22.5321	23.0911
HCN:HF	31.1699	31.2180
NCH:FH	12.2561	12.2948
HCN:HCN	19.5007	19.5358
1-naphtol:water	29.8641	29.7878

Table S4. The negative of the interaction energy data from Figures 1 and 2 of the main text. All values are in kJ/mol.

type of system	description	canonical CCSD(T)/CBS	focal-point DLPNO-CCSD(T)/CBS	DLPNO-CCSD(T)/CBS (aTZ→a5Z)
from the Set22 (the dimers S01–S22 are specified in Table S1)	S01	13.0305	13.0142	12.8962
	S02	20.6922	20.7372	20.5690
	S03	77.5433	77.8386	77.2000
	S04	66.5291	66.4772	65.8758
	S05	85.6371	85.0352	84.7209
	S06	70.2161	70.5020	70.2370
	S07	69.1001	68.2809	67.9070
	S08	2.1896	2.1279	2.1119
	S09	6.1332	6.0008	5.9369
	S10	5.8910	5.7626	5.6794
	S11	10.8905	9.8440	9.7653
	S12	17.4600	16.2025	16.0482
	S13	40.5791	38.6929	38.4389
	S14	19.0416	16.9231	16.8979
	S15	48.5693	46.2843	46.4699
	S16	6.2466	6.0974	6.0349
	S17	13.5639	13.2741	13.1535
	S18	9.5923	9.3236	9.2315
	S19	18.7783	18.5571	18.4302
	S20	11.2461	11.3192	11.1230
	S21	23.3231	23.1684	23.0930
	S22	29.3670	29.2546	29.2582
dispersion-dominated complex from Set3x6	aniline:methane	6.8409	6.4802	6.3846
	anisole:methane	7.3866	6.9824	6.8524
	1-naphtol:methane	9.1377	8.6859	8.5497
	1-naphtol:CO	8.3737	7.8983	8.1819
	1-naphtol:CO_2	12.6781	11.9629	11.9234
	anisole:anisole	27.1599	26.1590	26.1702
mixed-interactions complex from Set3x6	anisole:ammonia	12.0031	11.6934	11.7319
	1-naphtol:ethyne	16.9643	16.7201	16.4847
	HCl:HCl	7.9397	8.1103	7.8506
	benzene:water	13.4345	13.1087	12.9320
	anisole:CO_2	15.8595	15.4662	15.3480
	ethyne:ethyne	6.2616	6.1367	6.1881

electrostatics-dominated complex from Set3x6	1-naphtol:ammonia	40.5199	40.4598	40.2566
	HCl:water	22.5321	23.0911	22.5870
	HCN:HF	31.1699	31.2180	30.9987
	NCH:FH	12.2561	12.2948	12.2215
	HCN:HCN	19.5007	19.5358	19.4400
	1-naphtol:water	29.8641	29.7878	29.6252
furan:toluene stacked complex from ref. **[50]**	configuration #1	14.4349	13.6841	13.4646
	configuration #2	13.9410	12.9909	13.0000
	configuration #3	12.6215	11.9214	11.7363
	configuration #4	12.8154	12.0660	11.9985
	configuration #5	12.0621	11.3746	11.3712
	configuration #6	11.0006	10.3386	10.5337
	configuration #7	9.6457	9.4687	9.0669
miscellaneous	anthracene: cyclopropenium	88.8474	87.7087	87.0019
	pyridine:pyridine	15.8174	14.8574	14.7448

Table S5. The negative of the interaction energy data from Figure 3 of the main text. All values are in kJ/mol.

type of system	description	canonical CCSD(T)/CBS	approximated CCSD(T)/CBS
dispersion-dominated complex from Set3x6	aniline:methane	29.8641	29.4069
	anisole:methane	12.6781	11.8102
	1-naphtol:methane	6.8409	6.5375
	1-naphtol:CO	7.3866	7.0066
	1-naphtol:CO_2	31.1699	31.0802
	anisole:anisole	12.2561	11.9452
mixed-interactions complex from Set3x6	anisole:ammonia	27.1599	26.3823
	1-naphtol:ethyne	9.1377	8.6573
	HCl:HCl	8.3737	7.7178
	benzene:water	40.5199	40.3112
	anisole:CO_2	12.0031	11.7578
	ethyne:ethyne	16.9643	16.9575
electrostatics-dominated complex from Set3x6	1-naphtol:ammonia	7.9397	7.9303
	HCl:water	13.4345	13.2035
	HCN:HF	15.8595	15.2338
	NCH:FH	6.2616	6.3140
	HCN:HCN	22.5321	22.0560
	1-naphtol:water	19.5007	19.4882
furan:toluene stacked complex from ref. **[50]**	configuration #1	14.4349	13.6660
	configuration #2	13.9410	12.8667
	configuration #3	12.6215	12.0365
	configuration #4	12.8154	11.9090
	configuration #5	12.0621	11.3044
	configuration #6	11.0006	10.1508
	configuration #7	9.6457	8.1819
miscellaneous	anthracene: cyclopropenium	88.8474	87.1310
	pyridine:pyridine	15.8174	14.7422

Table S6. The negative of the interaction energy data from Figure 5 of the main text. All values are in kJ/mol.

furan:toluene configuration	canonical CCSD(T)/CBS	DLPNO-CCSD(T)/CBS	extrapolated (aDZ, aTZ)	DLPNO-CCSD(T)/ cc-pVQZ	SAPT-DFT/CBS
#1	14.4349	13.6841	13.6660	17.4885	14.5393
#2	13.9410	12.9909	12.8667	17.0631	14.2296
#3	12.6215	11.9214	12.0365	15.7320	12.9284
#4	12.8154	12.0660	11.9090	15.5797	13.1685
#5	12.0621	11.3746	11.3044	15.0651	12.4759
#6	11.0006	10.3386	10.1508	13.5870	11.4910
#7	9.6457	9.4687	8.1819	12.1771	10.1160

Table S7. Parameters of the linear regression for 7 configurations of the furan···toluene dimer described in the text; $\{y\} = a \times \{x\} + b$, where $\{x\}$ represents the canonical $\Delta E^{\mathrm{CBS}}_{\mathrm{CCSD(T)}}$ data and σ is the standard deviation of residuals. The $\{x\}$, $\{y\}$ dataset are available from Table S6.

method to obtain dataset $\{x\}$	a	b (in kJ/mol)	σ (in kJ/mol)	adjusted R^2
DLPNO-CCSD(T)/CBS	0.8780	0.839	0.123	0.9913
extrapolated (aDZ, aTZ)	1.126	1.33	0.433	0.9931
DLPNO-CCSD(T)/ cc-pVQZ	1.091	–2.04	0.427	0.9789
SAPT-DFT/CBS	0.9296	1.22	0.105	0.9981
